# Small-Spored *Alternaria* Species (Pleosporales, Pleosporaceae) Associated with Cucurbitaceae in China

**DOI:** 10.3390/jof12030201

**Published:** 2026-03-10

**Authors:** Lin He, Pingping Sun, Zhengnan Li, Jianxin Deng, Sein Lai Lai Aung

**Affiliations:** 1 Department of Plant Protection, College of Agriculture, Yangtze University, Jingzhou 434025, China; helin3022@hotmail.com; 2 MARA Key Laboratory of Sustainable Crop Production in the Middle Reaches of the Yangtze River (Co-Construction by Ministry and Province), Yangtze University, Jingzhou 434025, China; 3 College of Horticulture and Plant Protection, Inner Mongolia Agricultural University, Hohhot 010018, China; sunpingping@imau.edu.cn (P.S.); lizhengnan@imau.edu.cn (Z.L.)

**Keywords:** Cucurbitaceae, small-spored *Alternaria*, taxonomy, novel species, new records

## Abstract

Cucurbitaceous plants comprise a wide range of economically important vegetable and fruit crops. In this study, *Alternaria* species associated with Cucurbitaceae were investigated using an integrative approach combining multilocus phylogenetic analyses and morphological characterization. Two novel species, *Alternaria charantiicola* sp. nov. and *A. cucumicola* sp. nov., were identified from *Momordica charantia* and *Cucumis* sp., respectively. In addition, five *Alternaria* species, namely *A. zeae*, *A. lycopersici*, *A. sanguisorbae*, *A. pulvinifungicola*, and *A. solanicola*, are reported for the first time from cucurbitaceous hosts. These findings expand the known species diversity of *Alternaria* associated with Cucurbitaceae in China and provide a taxonomic basis for the accurate diagnosis of *Alternaria*-related diseases in cucurbit crops.

## 1. Introduction

Cucurbitaceae is one of the most important families of food plants and is commonly referred to as the cucurbits or the gourd family [1]. In terms of agricultural importance, it ranks after Poaceae, Fabaceae, and Solanaceae [2]. The family comprises approximately 115 genera and 960 species, which are mainly distributed in tropical and subtropical regions, with a few species extending into temperate areas [3]. In China, Cucurbitaceae includes about 32 genera and 154 species, predominantly distributed in southern and southwestern regions, with some species also occurring in northern China [4]. Cucurbitaceous plants encompass a wide range of economically important vegetable and fruit crops, including cucumber (*Cucumis sativus*), pumpkin (*Cucurbita pepo*, *C. moschata*, *C. argyrosperma*), wax gourd (*Benincasa hispida*), and bitter gourd (*Momordica charantia*), as well as fruit crops such as watermelon (*Citrullus lanatus*), melon (*C. melo*), and monk fruit (*Siraitia grosvenorii*) [1,5]. Among them, *M. charantia* and *S. grosvenorii* are notable for their combined culinary and medicinal value and have traditionally been used for cough relief, heat alleviation, regulation of intestinal function, and strengthening of the spleen and stomach [5]. However, the frequent occurrence and rapid spread of diseases have become a major bottleneck constraining the sustainable development of Cucurbitaceae crop production. In particular, fungal diseases cause severe yield losses in cucurbit crops, including powdery mildew caused by *Erysiphe cichoracearum*, downy mildew caused by *Pseudoperonospora cubensis*, Fusarium wilt caused by *Fusarium oxysporum*, and root rot caused by *F. solani* [5]. Notably, the genus *Alternaria* has been widely reported from Cucurbitaceae, including *A. alternata* [6], *A. baoshanensis* [7], *A. brassicae* [8], *A. caudata* [8], *A. cucumericola* [8], *A. cucumerina* [6], *A. gaisen* [8], *A. granulosa* [8], *A. hydrangea* [9], *A. jingzhouensis* [10], *A. infectoria* [8], *A. loofahae* [8], *A. momordicae* [10], *A. nigrescens* [8], *A. peponicola* [8], *A. peponis* [8], and *A. tenuissima* [6].

The genus *Alternaria* was established by Nees in 1816 and typified by *A. tenuis* [11]. It ranks among the top ten most cited fungal genera and is associated with more than 4000 host plant species worldwide [7,12,13,14]. To date, over 400 species have been recognized within *Alternaria*, encompassing saprophytic, endophytic, and phytopathogenic lifestyles [12,13,15,16]. Historically, species delimitation in *Alternaria* relied primarily on morphological characteristics, including colony features, conidial morphology, and sporulation patterns [8]. Based on conidial size, the genus was traditionally divided into large-spored taxa (60–100 μm) and small-spored taxa (<60 μm). However, the taxonomy of small-spored *Alternaria* has long been problematic owing to their highly conserved and overlapping morphological traits [8]. In recent decades, DNA-based molecular approaches have greatly improved the resolution of phylogenetic relationships within *Alternaria* [7,13,14]. Early multilocus analyses tended to cluster many small-spored taxa in section *Alternaria*, containing 11 species and one species complex [14]. With the continued discovery and description of new small-spored species, taxonomic boundaries within section *Alternaria* have become increasingly refined, and species delimitation has gradually become clearer. To date, more than 30 phylogenetically distinct species have been accepted within this section, highlighting the previously underestimated diversity and complexity of this group [7,10,15,16,17,18]. Recent studies have shown that many taxa within section *Alternaria* form species complexes characterized by subtle morphological differentiation yet distinct multilocus phylogenetic lineages [7,15]. The integration of multilocus datasets with emerging phylogenomic evidence has progressively refined species boundaries, uncovering extensive cryptic diversity among small-spored *Alternaria* [14,19].

In this study, *Alternaria* species associated with Cucurbitaceae in China were examined through a comprehensive taxonomic investigation. An integrative approach combining multilocus phylogenetic analyses with morphological characterization was applied, leading to the recognition of two novel species and five newly recorded species from symptomatic cucurbitaceous leaves. This study establishes a taxonomic baseline for understanding the diversity of *Alternaria* associated with Cucurbitaceae.

## 2. Materials and Methods

### 2.1. Sampling and Isolation

Plants exhibiting symptoms resembling *Alternaria* leaf spot or blight were collected from three Cucurbitaceae genera (*Citrullus*, *Cucumis*, and *Momordica*) across six provinces in China (Hainan, Heilongjiang, Hubei, Guangxi, Jilin, and Liaoning) during 2016–2024. Symptomatic tissues were excised into small fragments (approximately 2 × 2 mm) using sterile blades and placed on moist filter paper in Petri dishes, followed by incubation at 25 °C in the dark. Single conidia were examined under a stereomicroscope and aseptically transferred onto potato dextrose agar (PDA: Difco, Montreal, QC, Canada) to obtain pure cultures. All plant specimens and fungal isolates were deposited in the Fungal Herbarium of Yangtze University (Jingzhou, China). Dried cultures of isolates were preserved in the same collection for long-term storage.

### 2.2. Morphological Characteristics

Colony characteristics were examined on 90 mm PDA plates incubated at 25 °C in the dark for 7 days. Conidial morphology, including size, shape, and sporulation patterns, was assessed on potato carrot agar (PCA) and V8 juice agar (V8A) following incubation at 22 °C under an 8 h light/16 h dark photoperiod. After 7 days, conidia and sporulation structures were observed and photographed using a Nikon Eclipse Ni-U microscope (Nikon, Tokyo, Japan). Conidial dimensions were measured from 50 randomly selected conidia per isolate.

### 2.3. DNA Extraction, PCR Amplification, and Sequencing

Fresh mycelia of each isolate were harvested from 5–7-day-old colonies grown on PDA. Genomic DNA was extracted using a modified CTAB method [20]. Seven gene fragments, including the internal transcribed spacer (ITS) region of rDNA, glyceraldehyde-3-phosphate dehydrogenase (*GAPDH*), translation elongation factor 1-alpha (*TEF1*), RNA polymerase II second largest subunit (*RPB2*), *Alternaria* major allergen gene (*Alt a 1*), endopolygalacturonase (*EndoPG*) gene, and an anonymous gene region (OPA10-2), were amplified using the primer pairs ITS5/ITS4 [21], gpd1/gpd2 [22], EF1-728F/EF1-986R [23], RPB2-5F/RPB2-7cR [24], Alt-for/Alt-rev [25], PG3/PG2b [26], and OPA10-2L/OPA10-2R [26], respectively. PCR amplifications were performed in a Bio-Rad T100™ Thermal Cycler (Bio-Rad, Hercules, CA, USA) using a total reaction volume of 25 μL, which consisted of 21 μL of 1.1× Taq PCR Star Mix (TSINGKE Co., Ltd., Beijing, China), 1 μL of each primer (10 μM), and 2 μL of template DNA. The PCR cycling conditions followed those described previously [18]. PCR products were verified by electrophoresis on 1% agarose gels and sequenced commercially by TSINGKE (Beijing, China). All newly generated sequences were deposited in GenBank (https://www.ncbi.nlm.nih.gov/), with accession numbers provided in Table 1.

### 2.4. Phylogenetic Analysis

Nucleotide sequences generated in this study were first subjected to BLASTn searches against the NCBI database (https://www.ncbi.nlm.nih.gov/; accessed on 23 January 2026) to assess sequence similarity. Reference sequences of *Alternaria* species used for phylogenetic analyses were retrieved from GenBank (Table 1). Phylogenetic analyses were conducted using the One-click Fungal Phylogenetic Tool (OFTP: v1.9.0) [27]. Sequence alignments were performed with MAFFT v7.520 [28] and subsequently trimmed using TrimAl v1.2 [29]. ModelFinder v1.6.12 [30] was used to evaluate and select the best-fitting nucleotide substitution models for each dataset based on the Bayesian information criterion (BIC). Phylogenetic trees were inferred using maximum likelihood (ML) and Bayesian inference (BI) methods based on concatenated, partitioned datasets. ML analyses were performed in IQ-TREE v1.6.12 [31] with 1000 bootstrap replicates to assess branch support. BI analyses were carried out in MrBayes v3.2.7 [32] using four Markov chain Monte Carlo (MCMC) chains run for 50,000,000 generations, sampling every 100 generations. In addition, for the BI analyses, the first 25% of trees were discarded as burn-in, and posterior probabilities were calculated from the remaining trees. The BI consensus tree was generated once the average standard deviation of split frequencies dropped below 0.01.

## 3. Results

### 3.1. Fungal Isolates

In total, 79 *Alternaria* isolates were obtained, and 13 representative isolates were selected for further study based on observed morphological diversity and preliminary ITS sequence comparisons.

### 3.2. Phylogenetic Analysis

**Figure 1:**
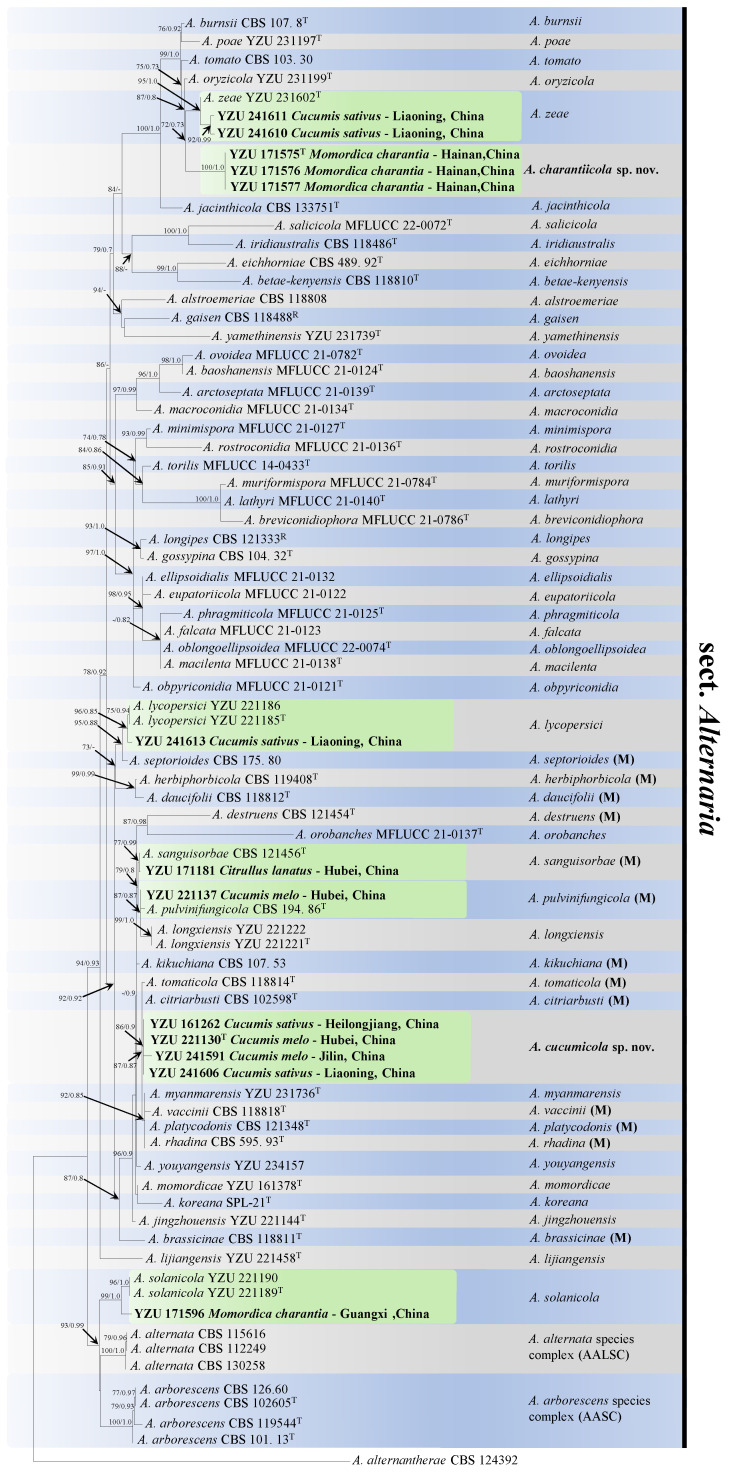
Maximum likelihood phylogenetic tree inferred from concatenated sequences of ITS, *GAPDH*, *RPB2*, *TEF1*, *Alt a 1*, *EndoPG*, and OPA10-2 for *Alternaria* spp. Bootstrap support values (BS) and Bayesian posterior probabilities (PP) are shown at the nodes (BS/PP). Strains obtained in this study are indicated in bold. **Ex-type strains are marked with “T”, representative strains with “R”, and morphospecies with “(M)”**. *Alternaria alternantherae* CBS 124392 was used as the outgroup.

Based on BLASTn searches, all isolates obtained in this study were assigned to *Alternaria* section *Alternaria*. In total, 84 strains were included in the phylogenetic analyses, including 13 representative small-spored isolates obtained in this study, with *A. alternantherae* CBS 124392 selected as the outgroup. The concatenated dataset comprised seven loci (ITS, *GAPDH*, *TEF1*, *RPB2*, *Alt a 1*, *EndoPG*, and OPA10-2), yielding an alignment of 3399 characters, of which 9.26% were missing. The individual gene regions contributed 513 bp (ITS), 576 bp (*GAPDH*), 210 bp (*TEF1*), 555 bp (*RPB2*), 471 bp (*Alt a 1*), 444 bp (*EndoPG*), and 630 bp (OPA10-2). The best-fit substitution models were K2P (ITS), TNe + G4 (*GAPDH*), K2P (*TEF1*), TNe + I (*RPB2*), K2P + G4 (*Alt a 1*), K3P + I (*EndoPG*), and K2P + G4 (OPA10-2). Maximum likelihood (ML) and Bayesian inference (BI) analyses produced congruent topologies; the ML tree is shown in Figure 1. Phylogenetic reconstruction resolved the examined isolates into seven distinct clades within section *Alternaria*. Isolates YZU 241610 and YZU 241611, obtained from *C. sativus*, clustered with *A. zeae* YZU 231602 with strong support (BS = 95%, PP = 1.00). In contrast, isolates YZU 171575, YZU 171576, and YZU 171577 from *M. charantia* formed a well-supported (BS = 72%, PP = 0.73), independent lineage closely related to *A. zeae*, representing a distinct novel clade. Isolate YZU 241613 (*C. sativus*) grouped with *A. lycopersici* (BS = 96%, PP = 0.85). Meanwhile, isolates YZU 171181 from *C. lanatus* and YZU 221137 from *C. melo* clustered within the same clade (BS = 79%, PP = 0.8), forming sister relationships with *A. sanguisorbae* CBS 121456 (BS = 77%, PP = 0.99) and *A. pulvinifungicola* CBS 194.86, respectively. Notably, isolates YZU 161262 (*C. sativus*), YZU 221130 (*C. melo*), YZU 241591 (*C. melo*), and YZU 241606 (*C. sativus*) formed a strongly supported monophyletic group (BS = 86%, PP = 0.90), clearly separated from all previously described species (BS = 87%, PP = 0.87), representing another novel phylogenetic lineage. Isolate YZU 171596 clustered with *A. solanicola* with strong support (BS = 99%, PP = 1.00). In summary, all isolates obtained in this study belong to section *Alternaria*, comprising two novel phylogenetic clades and five clades corresponding to previously described species (Figure 1).

### 3.3. Taxonomy

**Figure 2:**
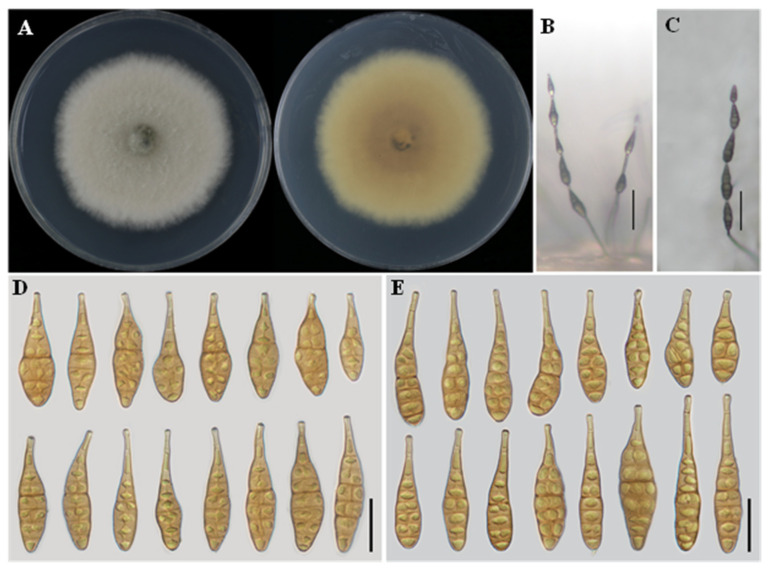
Morphology of *Alternaria charantiicola* sp. nov. (YZU 171577). (**A**) Colony on PDA for 7 days at 25 °C; (**B**) sporulation on PCA; (**C**) sporulation on V8A; (**D**) Conidia on PCA; (**E**) Conidia on V8A. Scale bars: 50 μm (**B**,**C**); 25 μm (**D**,**E**).

***Alternaria charantiicola*** **L. He, S.L.L. Aung and J.X. Deng, sp. nov. (Figure 2).**

MycoBank No.: 862358.

**Etymology:** The specific epithet *charantiicola* refers to the host plant *Momordica charantia*, from which the fungus was isolated.

**Typification:** China, Hainan Province, Haikou City; diseased leaves of *M. charantia*, 2017, J.X. Deng; Holotype: YZU-H-2017075; Ex-type culture: YZU 171575.

**Description:** Colonies on PDA cottony, white with filamentous margins, reverse rosy buff to vinaceous buff at the center, 56–57 mm in diameter. On PCA, conidiophores solitary, arising from the substrate, simple, straight or flexuous, 19–85 × 2–4 μm, with 2–3 septa. Conidiogenous cells terminal, determinate, cylindrical, apically doliiform, 6–15 × 2–4 μm, with 1–2 conidiogenous loci. Conidia in chains of 4–6 units, arising from the apex or near the apex of conidiophores or terminal hyphae, muriform, ellipsoidal to flask-shaped, rostrate, body 36–54 × 11–15 μm, with 2–5 transverse septa; apical beak 3–9 × 2–3 μm. On V8A, conidiophores simple, straight or flexuous, 39–55 × 2–5 μm. Conidiogenous cells terminal, determinate, cylindrical, apically doliiform, 5–14 × 3–4 μm, with a single conidiogenous locus, sometimes slightly swollen near the locus. Conidia in chains of 3–5 units, unbranched, muriform, ellipsoidal to flask-shaped, rostrate, body 39–55 × 11–18 μm, with 2–5 transverse septa; apical beak 7–21 × 2–3 μm.

**Notes:** Phylogenetic analyses based on a concatenated dataset of seven loci unambiguously placed *A. charantiicola* within a clade comprising *A. zeae* YZU 231602 and *A. oryzicola* YZU 231199. Nucleotide sequence comparisons showed that strain YZU 171575 differed from the representative strain of *A. zeae* by 2 bp in *Alt a 1*, 22 bp in OPA10-2, 1 bp in *RPB2*, and 10 bp in *TEF1*. In comparison with *A. oryzicola*, a total of 33 bp differences were detected, including 2 bp in *Alt a 1*, 19 bp in OPA10-2, 2 bp in *RPB2*, and 10 bp in *TEF1*. Morphologically, isolate YZU 171575 is further distinguished from *A. zeae* and *A. oryzicola* by its larger conidial body size (Table 2). Taken together, the concordant molecular and morphological evidence supports the recognition of isolate YZU 171575 as a distinct species, herein described as *A. charantiicola* sp. nov.

**Figure 3:**
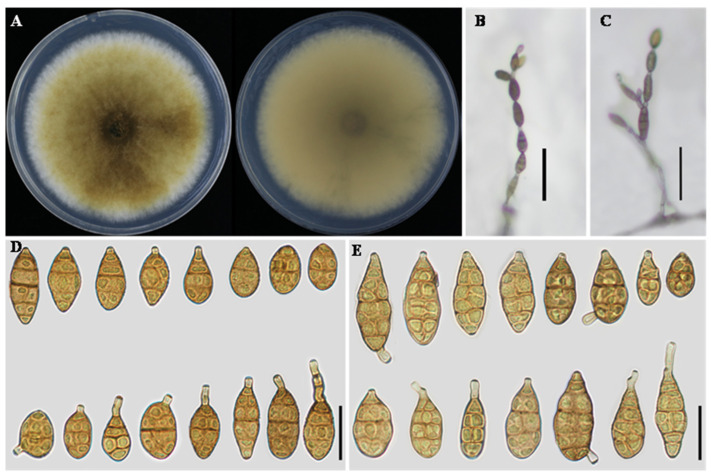
Morphology of *Alternaria cucumicola* sp. nov. (YZU 221130). (**A**) Colony on PDA for 7 days at 25 °C; (**B**) sporulation on PCA; (**C**) sporulation on V8A; (**D**) Conidia on PCA; (**E**) Conidia on V8A. Scale bars: 50 μm (**B**,**C**); 25 μm (**D**,**E**).

***Alternaria cucumicola*** **L. He, S.L.L. Aung and J.X. Deng, sp. nov. (Figure 3).**

MycoBank No.: 862357.

**Etymology:** The specific epithet *cucumicola* refers to the host genus *Cucumis* (*C. sativus* and *C. melo*), from which the fungus was isolated.

**Typification:** China, Hubei Province, Wuxue City; diseased leaves of *C. melo*, 2022, J.X. Deng; Holotype: YZU-H-2022029; Ex-type culture: YZU 221130.

**Additional specimens examined (China):** Heilongjiang Province, on *C. sativus*, YZU 161262, 2016; Jilin Province, Changchun City, on *C. melo*, YZU 241591, 2024; Liaoning Province, Jinzhou City, on *C. sativus*, YZU 241606, 2024.

**Description:** Colonies on PDA circular, velvety to cottony, pale brown to light olivaceous brown, darker at the center, with a narrow whitish margin; reverse pale yellow to light brown, 60–65 mm in diameter. On PCA, conidiophores arising from the substrate or from aerial hyphae, erect to slightly flexuous, mostly unbranched, occasionally geniculate near the apex, 30–80 × 3–5 μm. Conidiogenous cells integrated, terminal, apically doliiform, with a single conidiogenous locus. Conidiation predominantly acrogenous, with conidia produced successively from the apical beak of the terminal conidium; the apical beak may develop multiple conidiogenous loci, giving rise to short conidial chains. Conidia in chains of 2–6 units, ellipsoid to narrow-obclavate or obclavate, 18–42 × 9–16 μm, with 2–5 transverse septa. Apical beak absent to short, usually 3–15 μm long. On V8A, conidiophores similar in morphology to those on PCA, 25–65 × 3–5 μm. Conidiation both acrogenous and intercalary; in addition to apical conidiation, lateral conidia were frequently observed arising from the beak or body of intercalary conidia within the chain, resulting in branched or irregular conidial chains. Conidia solitary or in chains of 3–6 units, ellipsoid to obclavate, 20–45 × 10–18 μm, with 2–5 transverse septa. Apical beak short or absent, 2–12 μm long.

**Notes:** Phylogenetic analyses based on the combined multilocus dataset placed *A. cucumicola* within a clade comprising *A. citriarbusti* CBS 102598, *A. platycodonis* CBS 121348, *A. rhadina* CBS 595.93, *A. tomaticola* CBS 118814, *A. vaccinii* CBS 118818, and *A. myanmarensis* YZU 231736. Within this clade, *A. cucumicola* was resolved as a clearly independent lineage, strongly supported (BS = 99%, PP = 1.00). Phylogenetically, *A. platycodonis*, *A. rhadina*, and *A. myanmarensis* were identical. *A. cucumicola* differs from these taxa at two loci, with 1 bp in *GAPDH* and 3 bp in *EndoPG* (including two gaps). It also differs from *A. citriarbusti* by 1 bp in *RPB2* and 2 bp in *EndoPG*, and from *A. tomaticola* by 2 bp in *Alt a 1* and 1 bp in *EndoPG*. Morphologically, isolate YZU 221130 is distinguished from closely related taxa by its conidial body size, shorter conidial chains, and the absence of a thin, elongated apical beak (Table 2). Collectively, the evidence supports the delimitation of isolate YZU 221130 as a distinct species.

**Figure 4:**
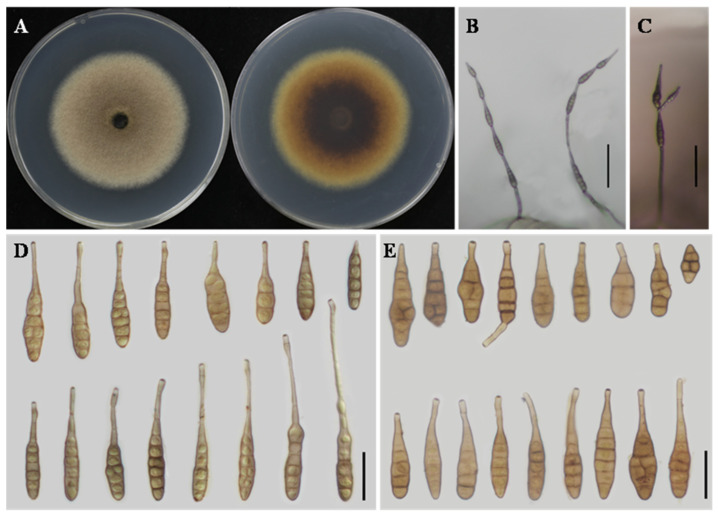
Morphology of *Alternaria zeae* (YZU 241610). (**A**) Colony on PDA for 7 days at 25 °C; (**B**) sporulation on PCA; (**C**) sporulation on V8A; (**D**) Conidia on PCA; (**E**) Conidia on V8A. Scale bars: 50 μm (**B**,**C**); 25 μm (**D**,**E**).

***Alternaria zeae*** **H.F. Liu and J.X. Deng, MycoKeys 116: 167. 2025 (Figure 4).**

**Additional isolates examined:** China, Liaoning Province, Jinzhou City, Linghe District; diseased leaves of *C. sativus*; collected in 2024 by J.X. Deng. Living culture deposited as YZU 241610 and YZU 241611.

**Description:** Colonies on PDA cottony, pale vinaceous grey, with filamentous margins; reverse vinaceous grey with buff filamentous margins, 61–62 mm in diameter. On PCA, conidiophores solitary, arising directly from the substrate, simple, flexuous, 24–149 × 2–3 μm, with 2–6 septa. Conidiogenous cells terminal, determinate, cylindrical, smooth, thin-walled, apically doliiform, 2–5 × 2–4 μm, with a single conidiogenous locus, cicatrized at conidial secession. Conidia borne in unbranched chains of 4–6 units, arising from the apex or near the apex of conidiophores or terminal hyphae, muriform, ovate, ellipsoid to flask-shaped, rostrate; conidial body 28–42 × 9–17 μm, with 1–6 transverse septa; apical beak 4–26 (–50) × 2–3 μm. On V8A, conidiophores simple, straight to flexuous, 19–65 × 2–4 μm, with 2–3 septa. Conidiogenous cells terminal, determinate, cylindrical, smooth, thin-walled, apically doliiform, 5–12 × 2–4 μm, with a single conidiogenous locus, sometimes slightly swollen near the conidiogenous locus. Conidia borne in chains of 4–6 units, arising from the apex or near the apex of conidiophores or terminal hyphae, muriform, ellipsoid to flask-shaped, rostrate; conidial body 27–45 × 8–16 μm, with 1–6 transverse septa; apical beak 3–16 × 2–3 μm, occasionally elongating to form a secondary conidiophore; chains unbranched.

**Notes:** *A. zeae* was originally described from *Zea mays* [16]. In the present study, multilocus phylogenetic analyses revealed that isolates YZU 241610 and YZU 241611 clustered with *A. zeae* with strong support (BS = 95%, PP = 1.00) (Figure 1). Based on both phylogenetic evidence and morphological characteristics, these isolates are identified as *A. zeae*. This study represents a new host record of *A. zeae* on *C. sativus*.

**Figure 5:**
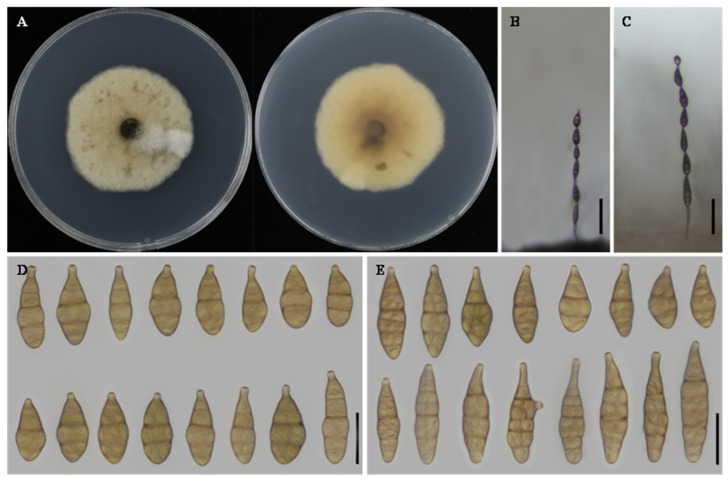
Morphology of *Alternaria lycopersici* (YZU 241613). (**A**) Colony on PDA for 7 days at 25 °C; (**B**) sporulation on PCA; (**C**) sporulation on V8A; (**D**) Conidia on PCA; (**E**) Conidia on V8A. Scale bars: 50 μm (**B**,**C**); 25 μm (**D**,**E**).

***Alternaria lycopersici*** **Y.N. Gou and J.X. Deng, J. Fungi. 9: 800. 2023 (Figure 5).**

**Additional isolates examined:** China, Liaoning Province, Jinzhou City, Linghe District; diseased leaves of *C. sativus*; collected in 2024 by J.X. Deng. Living culture deposited as YZU 241613.

**Description:** Colonies on PDA cottony, light cottony, and buff in the center, reverse villiform with white at the edge, 42–44 mm in diameter. On PCA, conidiophores solitary, arising from the substrate, simple, flexuous, 42–81 × 3–5 μm, with 2–5 septa. Conidiogenous cells terminal, apically doliiform, 5–10 × 2–5 μm, with a single conidiogenous locus. Conidia borne in chains of 5–7 units, arising from the apex or near the apex of conidiophores or terminal hyphae, muriform, clavate, ovoid, or long ellipsoid to flask-shaped, rostrate; conidial body 25–42 × 11–17 μm, with 1–4 transverse septa. On V8A, conidiophores solitary, arising from the substrate, simple, flexuous, 50–67 × 2–5 μm, with 2–5 septa. Conidiogenous cells terminal, apically doliiform, 5–10 × 2–5 μm, with a single conidiogenous locus. Conidia borne in chains of 5–7 units, arising from the apex or near the apex of conidiophores or terminal hyphae, muriform, clavate, long ellipsoid or ovoid, rostrate; conidial body 16–53 × 10–15 μm, with 1–4 transverse septa; chains unbranched.

**Notes:** *A. lycopersici* was previously known only from *Solanum lycopersicum* [17]. In the present study, isolate YZU 241613 was recovered from diseased leaves of *C. sativus*. Phylogenetic inference based on seven concatenated loci placed this isolate within the *A. lycopersici* clade with strong statistical support (BS = 96%, PP = 0.85). Its morphological features were also in agreement with the original description of *A. lycopersici* [17]. Accordingly, *C. sativus* is herein reported as a new host for *A. lycopersici*.

**Figure 6:**
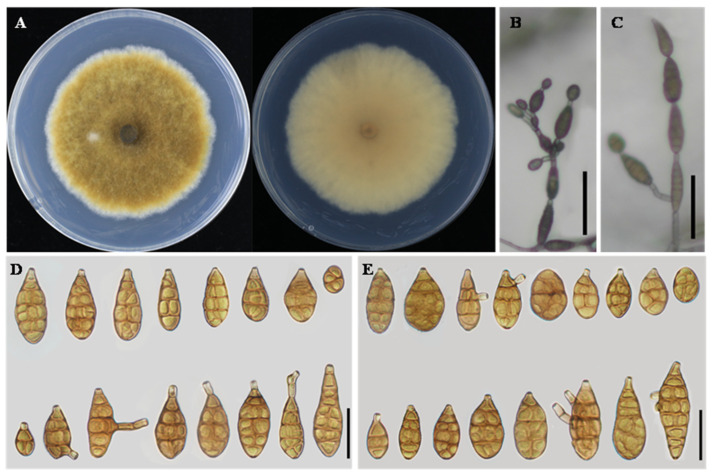
Morphology of *Alternaria sanguisorbae* (YZU 171181). (**A**) Colony on PDA for 7 days at 25 °C; (**B**) sporulation on PCA; (**C**) sporulation on V8A; (**D**) Conidia on PCA; (**E**) Conidia on V8A. Scale bars: 50 μm (**B**,**C**); 25 μm (**D**,**E**).

***Alternaria sanguisorbae*** **M.X. Gao and T.Y. Zhang, Mycosystema 19: 456. 2000 (Figure 6).**

**Additional isolates examined:** China, Hubei Province, Wuxue City; diseased leaves of *C. lanatus*; collected in 2017 by J.X. Deng. Living culture deposited as YZU 171181.

**Description:** Colonies on PDA honey-colored, with white filamentous margins on the surface; reverse buff with filamentous margins, 55–60 mm in diameter. On PCA, conidiophores solitary, arising directly from the substrate, simple, flexuous, 45–80 × 3–5 μm, with 4–6 septa. Conidiogenous cells terminal, 3–4 × 3–5 μm, with a single conidiogenous locus, cicatrized after conidial secession. Conidiation predominantly acrogenous: conidia produced successively from the apical beak of terminal conidia; the beak may develop multiple conidiogenous loci, resulting in short conidial chains. Conidia in chains of 3–7 units, unbranched to slightly branched, ovoid to ellipsoid; conidial body 20–35 × 9–14 μm, with 4–6 transverse septa; apical beak 5–21 × 2–3 μm. On V8A, conidiophores solitary, 35–82 × 3–5 μm, with 4–5 septa. Conidiogenous cells 3–4 × 3–5 μm, with a single conidiogenous locus, cicatrized after conidial secession. Conidiation both acrogenous and intercalary; in addition to apical conidiation, lateral conidia frequently arise from the beak or body of intercalary conidia within the chain, giving rise to branched or irregular conidial chains. Conidia in chains of 3–7 units, ovoid to ellipsoid; conidial body 18–45 × 9–13 μm, with 4–6 transverse septa; apical beak 5–20 × 2–3 μm.

**Notes:** *A. sanguisorbae* was previously redefined as *A. alternata* by Woudenberg et al. [14], and was originally described from *Sanguisorba officinalis* [33]. In the present study, isolate YZU 171181 from *C. lanatus* clustered with *A. sanguisorbae* in the multilocus phylogeny. Accordingly, this isolate is reassigned to *A. sanguisorbae*, representing a new host record.

**Figure 7:**
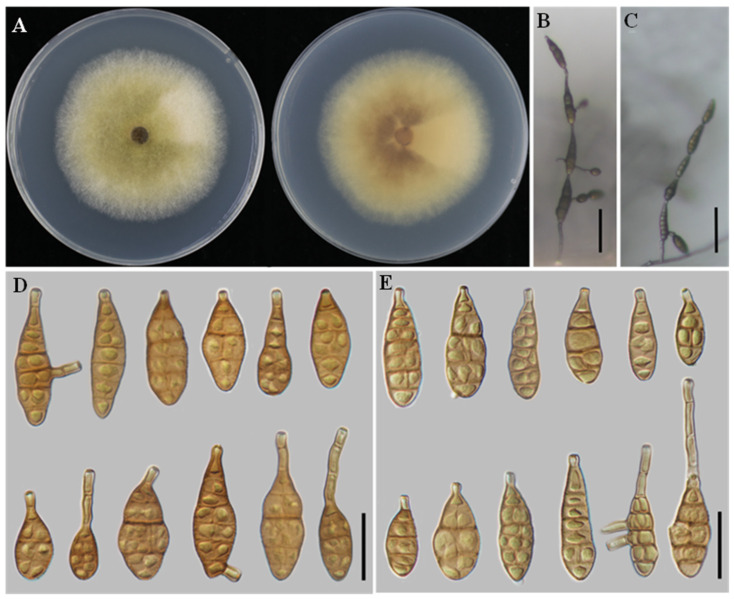
Morphology of *Alternaria pulvinifungicola* (YZU 221137). (**A**) Colony on PDA for 7 days at 25 °C; (**B**) sporulation on PCA; (**C**) sporulation on V8A; (**D**) Conidia on PCA; (**E**) Conidia on V8A. Scale bars: 50 μm (**B**,**C**); 25 μm (**D**,**E**).

***Alternaria pulvinifungicola*** **E.G. Simmons, CBS Biodiversity Ser. (Utrecht) 6: 514. 2007 (Figure 7).**

**Additional isolates examined:** China, Hubei Province, Jingzhou City; diseased leaves of *C. melo*; collected in 2022 by J.X. Deng. Living culture deposited as YZU 221137.

**Description:** Colonies on PDA olivaceous buff, with white sectoring and filamentous margins on the surface; reverse vinaceous buff with filamentous margins, 55–56 mm in diameter. On PCA, conidiophores solitary, arising from the substrate, simple, flexuous, 45–92 × 3–5 μm, with 4–6 septa. Conidiogenous cells terminal, apically doliiform, 3–32 × 3–5 μm, with 1–2 conidiogenous loci, cicatrized at conidial secession. Conidia borne in chains of 2–6 units, arising from the apex or near the apex of conidiophores or terminal hyphae, ovoid to ellipsoid, or obclavate; conidial body 25–51 × 7–17 μm, with 1–4 transverse septa; apical beak 20–95 × 3–5 μm; chains occasionally branched, bearing 2–4 conidia. On V8A, conidiophores solitary, 19–26 × 3–5 μm, with 1–4 septa. Conidiogenous cells terminal, 3–14 × 3–5 μm, with 1–2 conidiogenous loci. Conidia borne in chains of 2–6 units, arising from the apex or near the apex of conidiophores or terminal hyphae, ovoid to ellipsoid; conidial body 25–40 × 9–13 μm, with 1–6 transverse septa; apical beak 4–48 × 2–3 μm, occasionally developing into secondary conidiophores; chains branched.

**Notes:** *A. pulvinifungicola* was originally described from *Quercus* sp. [8], and was later treated as a synonym of *A. alternata* [14]. In the present study, isolate YZU 221137, obtained from *C. melo*, clustered with *A. pulvinifungicola* in the multilocus phylogenetic analysis. Based on this phylogenetic placement, the isolate is reassigned to *A. pulvinifungicola*, representing a new host record.

**Figure 8:**
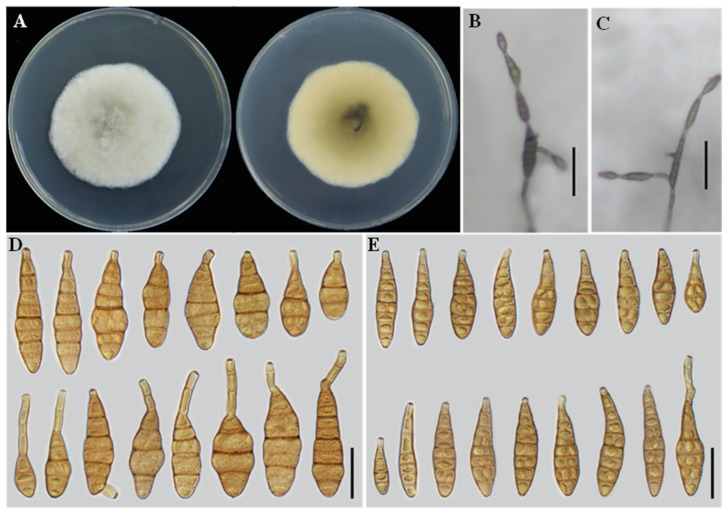
Morphology of *Alternaria solanicola* (YZU 171596). (**A**) Colony on PDA for 7 days at 25 °C; (**B**) sporulation on PCA; (**C**) sporulation on V8A; (**D**) Conidia on PCA; (**E**) Conidia on V8A. Scale bars: 50 μm (**B**,**C**); 25 μm (**D**,**E**).

***Alternaria solanicola*** **Y.N. Gou and J.X. Deng, J. Fungi. 9: 800. 2023 (Figure 8).**

**Additional isolates examined:** China, Guangxi Province, Nanning City; diseased leaves of *M. charantia*; collected in 2017 by J.X. Deng. Living culture deposited as YZU 171596.

**Description:** Colonies on PDA circular, cottony to floccose, white on the surface with a faint greyish-green center; reverse pale yellow to buff, 55–57 mm in diameter. On PCA, conidiophores solitary, arising from the substrate, straight to slightly flexuous, 25–70 × 3–5 μm. Conidia borne in chains of 2–4 units, short to long ovoid or ellipsoid; conidial body 20–50 × 7–14 μm, with 1–4 transverse septa and 0–2 longitudinal septa; beak absent or present, apical, 6–26 μm long. On V8A, conidiophores straight to slightly curved, septate, 18–65 × 3–5 μm. Conidia similar to those on PCA, produced in chains of 2–4 units, short to long ovoid or ellipsoid, 22–55 × 6–14 μm, with 1–4 transverse septa and 0–2 longitudinal septa.

**Notes:** *A. solanicola* was originally described from *S. lycopersicum* [17]. In the present study, isolate YZU 171596 recovered from *M. charantia* was placed within the *A. solanicola* clade in the multilocus phylogeny. Its morphological characteristics also correspond to those reported for this species. On this basis, YZU 171596 is regarded as *A. solanicola*, representing a new host record on *M. charantia*.

**Table 2:** Conidial morphology of *Alternaria* spp. from this study and previous publications.

Species	Conidia			Conidia per Chain	Substrate	References
	Shape	Body (μm)	Septa			
***A. charantiicola*** ** sp. nov**	**ellipsoidal to flask-shaped**	**36–54 × 11–15 (av.: 47 × 14)**	**2–5**	**4–6**	**PCA**	**This study**
		**39–55 × 11–18 (av.: 49 × 15.5)**	**2–5**	**3–5**	**V8A**	
*A. citriarbusti*	long-ellipsoid, narrow-ovoid, or narrowly ellipsoid	30–60 × 8–12	6–11	5–8	PCA	[34]
		50–80 × 10–12	7–9 (–11)	1–6	V8A	
***A. cucumicola*** ** sp. nov**	**ellipsoid to narrow-obclavate or obclavate**	**18–42 × 9–16 (av.: 26 × 13)**	**2–5**	**2–6**	**PCA**	**This study**
		**20–45 × 10–18 (av.: 28 × 14)**	**2–5**	**3–6**	**V8A**	
*A. lycopersici*	clavate, long ellipsoid or ovoid	18–41 × 9.5–13 (av.: 31 × 11)	1–7	2–4	PCA	[17]
		18.5–42.5 × 9–14 (av.: 30 × 9.5)	2–7	2–4	V8A	
		**25–42 × 11–17 (av.: 32 × 13)**	**1–4**	**5–7**	**PCA**	**This study**
		**16–53 × 10–15 (av.: 31.5 × 11)**	**1–4**	**5–7**	**V8A**	
*A. myanmarensis*	short to long ellipsoid or narrow-ovoid	10–30(–42) × 7–11	2–5	2–6	PCA	[18]
		8–29(–33) × 3–14	2–5	3–6	V8A	
*A. oryzicola*	narrow-obclavate, obclavate, or long ellipsoid	20–48 × 9–16	1–4	1–3	PCA	[16]
		18–56 × 9–16	2–6	1–3	V8A	
*A. platycodonis*	obclavata, ovoidea obpyriformia vel subellipsoidea	25.5–40 × 12–16	3–7	5–8	PCA	[33]
*A. pulvinifungicola*	long-ovoid, long-ellipsoid, or obclavate	30–40 × 8–12	4–8	8–12	PCA	[8]
		**25–51 × 7–17 (av.: 33 × 13)**	**1–4**	**2–6**	**PCA**	**This study**
		**25–40 × 9–13 (av.: 30 × 12)**	**1–6**	**2–6**	**V8A**	
*A. rhadina*	ovoid to narrow ovoid	35–45 × 8–16	4–7	9–15	Host	[35]
*A. sanguisorbae*	ovoid to ellipsoid or lemon-shaped	22–41.5 × 8–14	3–7	6–8	PCA	[33]
		**20–35 × 9–14 (av.: 28 × 12)**	**4–6**	**3–7**	**PCA**	**This study**
		**18–45 × 9–13 (av.: 31 × 12)**	**4–6**	**3–7**	**V8A**	
*A. solanicola*	short to long ovoid or ellipsoid	22–44 × 9–16.5 (av.: 32 × 12)	1–6	2–4	PCA	[17]
		22–43.5 × 9–16 (av.: 32.5 × 11.5)	1–6	2–4	V8A	
		**20–50 × 7–14 (av.: 33 × 12)**	**1–4**	**2–4**	**PCA**	**This study**
		**22–55 × 6–14 (av.: 34 × 10)**	**1–4**	**2–4**	**V8A**	
*A. tomaticola*	ellipsoid or ovoid	30–40 × 9–12 (larger conidia)	6–7	10–15	PCA	[8]
		12–25 × 7–13 (smaller conidia)	1–4			
*A. vaccinii*	ovoid or subellipsoid	15–50 × 7–9	1–8	8–10	PCA	[8]
*A. zeae*	ovate, ellipsoid or obclavate	26–46 × 10–18	3–6	1–4	PCA	[16]
		26–45 × 10–17	3–6	1–4	V8A	
		**28–42 × 9–17 (av.: 30 × 13)**	**1–6**	**4–6**	**PCA**	**This study**
		**27–45 × 8–16 (av.: 31.5 × 11)**	**1–6**	**4–7**	**V8A**	

**Notes:** Measurements for reference species were obtained from previously published descriptions based on type strains, whereas measurements for isolates examined in the present study were derived from representative isolates. Values obtained in this study are indicated in bold.

## 4. Discussion

In this study, *Alternaria* species associated with Cucurbitaceae were investigated across six provinces and three host genera in China. Using an integrative approach combining multilocus phylogenetic analyses with morphological characterization, two novel species, *A. charantiicola* sp. nov. and *A. cucumicola* sp. nov., together with five newly recorded species, were identified. Among these taxa, two previously described morphospecies, *A. sanguisorbae* and *A. pulvinifungicola*, were re-evaluated and taxonomically reassigned based on molecular phylogenetic evidence. Notably, the five newly recorded species had previously been reported only from non-cucurbit hosts, and their detection in this study extends their known host range, suggesting that some small-spored *Alternaria* species may exhibit relatively broad host associations or opportunistic colonization on cucurbit plants. Furthermore, *A. cucumicola* sp. nov. was consistently isolated from different cucurbit hosts across multiple geographic locations, indicating a potentially broad host association within Cucurbitaceae rather than strict host specificity. Although the isolates were obtained from symptomatic leaves, pathogenicity tests were not conducted; therefore, the taxa reported here should be regarded as disease-associated rather than confirmed pathogens, and their pathogenic roles require further verification. Overall, these findings expand current knowledge of host diversity within section *Alternaria* and provide a strengthened taxonomic framework for future systematic studies of *Alternaria* associated with cucurbit hosts.

All isolates obtained in the present study belong to the small-spored group within *Alternaria* section *Alternaria*. As noted above, the taxonomy of small-spored *Alternaria* has long been controversial owing to highly similar and overlapping morphological characteristics [15]. Based solely on morphological features, Simmons [8] recognized 128 small-spored species. However, subsequent molecular phylogenetic studies demonstrated that many of these taxa are phylogenetically indistinguishable and have largely been treated as *A. alternata* within section *Alternaria*, with the 35 morphospecies described by Simmons [8] subsequently subsumed into this complex [13,14]. This reclassification represented a pragmatic taxonomic solution at the time, given the limited molecular data available and the widespread reliance on morphology-based taxonomy. By consolidating poorly resolved morphospecies under *A. alternata*, it helped stabilize the taxonomy of section *Alternaria* and established *A. alternata* as a central reference taxon within the section. Nevertheless, with the increasing availability of multilocus phylogenetic data, the taxonomic status of several morphologically defined taxa within section *Alternaria* is being re-evaluated, as these analyses continue to reveal previously unrecognized lineages within the *A. alternata* species complex [7,15].

Two additional novel small-spored *Alternaria* species were identified in this study through an integrative analysis combining multilocus phylogeny and morphological characterization. One species, *A. charantiicola* sp. nov., formed a well-supported and clearly distinct lineage in the phylogenetic analyses, clustering with but phylogenetically separated from *A. zeae* and *A. oryzicola*. Nucleotide sequence comparisons revealed that *A. charantiicola* sp. nov. differs from *A. zeae* by 30 bp across four loci (*Alt a 1*, OPA10-2, *RPB2*, and *TEF1*) and from *A. oryzicola* by 33 bp across the four same loci. Morphologically, *A. charantiicola* sp. nov. is readily distinguished from these closely related taxa by its noticeably larger conidial body size (Table 2), further supporting its recognition as an independent species. The second novel species, *A. cucumicola* sp. nov., was resolved within a clade comprising six taxa, including five morphospecies (*A. tomaticola*, *A. citriarbusti*, *A. vaccinii*, *A. platycodonis*, and *A. rhadina*) that were previously synonymized under *A. alternata* [14], as well as the recently described species *A. myanmarensis* [18]. Phylogenetic analyses showed that, despite relatively limited nucleotide differences between *A. cucumicola* sp. nov. and its closest relatives, the species was consistently resolved as an independent lineage (Figure 1). In contrast, *A. platycodonis*, *A. rhadina*, and *A. myanmarensis* showed identical nucleotide sequences across the analyzed loci, suggesting that they may represent potential synonyms. Morphological characters further support the distinct status of *A. cucumicola* sp. nov. Its conidia are larger than those of *A. myanmarensis* but smaller than those of *A. citriarbusti* (Table 2). In addition, *A. cucumicola* produces markedly shorter conidial chains (2–6 conidia per chain) than *A. tomaticola* (10–15), *A. vaccinii* (8–10), and *A. rhadina* (9–15). The absence of a thin, elongated apical beak further distinguishes *A. cucumicola* sp. nov. from *A. platycodonis*. Taken together, these concordant phylogenetic and morphological differences support the recognition of *A. cucumicola* as a novel species. These results highlight that, in taxonomic classification of section *Alternaria*, morphospecies concepts should not be overlooked, as morphological distinctions remain essential for accurate species delimitation.

In addition to the two new species, five small-spored *Alternaria* species are reported here as new records from Cucurbitaceae hosts, namely *A. zeae*, *A. lycopersici*, *A. sanguisorbae*, *A. pulvinifungicola*, and *A. solanicola*. Multilocus phylogenetic analyses showed that the examined isolates clustered with reference strains of the corresponding taxa in well-supported clades, and their morphological characteristics were consistent with previously published descriptions. In particular, two isolates identified as *A. sanguisorbae* and *A. pulvinifungicola,* respectively, formed distinct phylogenetic lineages together with their reference strains. Although these species were previously treated as synonyms of *A. alternata* [14], our results support their interpretation as distinct phylogenetic lineages within section *Alternaria*. Therefore, we retain the morphospecies names *A. sanguisorbae* and *A. pulvinifungicola* to distinguish these taxa from the broader *A. alternata* species complex. These findings further suggest that morphospecies concepts remain useful in the taxonomy of section *Alternaria* when interpreted in combination with multilocus phylogenetic evidence. Moreover, the continued discovery and description of new taxa indicates that the taxonomy of small-spored *Alternaria* remains dynamic and may benefit from comprehensive reassessment as additional molecular datasets become available.

## 5. Conclusions

In conclusion, the present study examined *Alternaria* species associated with Cucurbitaceae using an integrative approach combining multilocus phylogenetic analyses and morphological characterization. Two novel species and five newly recorded species were identified and documented. These findings enhance current understanding of species diversity and host associations of *Alternaria* in Cucurbitaceae and provide a robust taxonomic basis for future systematic and diversity studies of *Alternaria* associated with cucurbit hosts.

## Figures and Tables

**Table 1:** GenBank accession numbers of *Alternaria* spp. used for phylogenetic analysis.

Species	Strain	ITS	*Alta1*	*GAPDH*	*RPB2*	*TEF1*	OPA10-2	*EndoPG*
*A. alstroemeriae*	CBS 118808	KP124296	KP123845	KP124153	KP124764	KP125071	KP124601	KP123993
*A. alternantherae*	CBS 124392	KC584179	KP123846	KC584096	KC584374	KC584633	–	–
*A. alternata*	CBS 112249	KP124338	KP123886	KP124192	KP124806	KP125114	KP124648	KP124039
*A. alternata*	CBS 115616	AF347031	AY563301	AY278808	KC584375	KC584634	KP124663	JQ811978
*A. alternata*	CBS 130258	KP124385	KP123933	KP124237	KP124855	KP125163	KP124698	KP124089
*A. arborescens*	CBS 119544^T^	KP124408	KP123955	JQ646321	KP124878	KP125186	KP124722	KP124112
*A. arborescens*	CBS 102605^T^	AF347033	AY563303	AY278810	KC584377	KC584636	KP124712	AY295028
*A. arborescens*	CBS 101.13^T^	KP124392	KP123940	KP124244	KP124862	KP125170	KP124705	KP124096
*A. arborescens*	CBS 126.60	KP124397	JQ646390	KP124249	KP124867	KP125175	KP124710	KP124101
*A. arctoseptata*	MFLUCC 21-0139^T^	–	OK236755	OK236702	OK236655	OK236608	–	–
*A. baoshanensis*	MFLUCC 21-0124^T^	MZ622003	OK236760	OK236706	OK236659	OK236613	–	–
*A. betae-kenyensis*	CBS 118810^T^	KP124419	KP123966	KP124270	KP124888	KP125197	KP124733	KP124123
*A. brassicinae*	CBS 118811^T^	KP124356	KP123904	KP124210	KP124824	KP125132	KP124667	KP124057
*A. breviconidiophora*	MFLUCC 21-0786^T^	MZ621997	OK236751	OK236698	OK236651	OK236604	–	–
*A. burnsii*	CBS 107.8^T^	KP124420	KP123967	JQ646305	KP124889	KP125198	KP124734	KP124124
***A. charantiicola*** ** sp. nov**	**YZU 171575**	**PX963225**	**PX975302**	**PX975315**	**PX975328**	**PX975367**	**PX975341**	**PX975354**
	**YZU 171576**	**PX972451**	**PX975303**	**PX975316**	**PX975329**	**PX975368**	**PX975342**	**PX975355**
	**YZU 171577**	**PX972452**	**PX975304**	**PX975317**	**PX975330**	**PX975369**	**PX975343**	**PX975356**
*A. citriarbusti*	CBS 102598^T^	KP124329	KP123878	KP124184	KP124797	KP125105	KP124638	KP124031
***A. cucumicola*** ** sp. nov**	**YZU 161262**	**PX963216**	**PX975300**	**PX975313**	**PX975326**	**PX975365**	**PX975339**	**PX975352**
	**YZU 221130**	**PX963233**	**PX975312**	**PX975319**	**PX975332**	**PX975371**	**PX975345**	**PX975358**
	**YZU 241591**	**PX963237**	**PX975307**	**PX975321**	**PX975334**	**PX975373**	**PX975347**	**PX975360**
	**YZU 241606**	**PX972453**	**PX975308**	**PX975322**	**PX975335**	**PX975374**	**PX975348**	**PX975361**
*A. daucifolii*	CBS 118812^T^	KC584193	KP123905	KC584112	KC584393	KC584652	KP124668	KP124058
*A. destruens*	CBS 121454^T^	AF278836	JQ646402	AY278812	KP124837	KP125145	KP124680	KP124071
*A. eichhorniae*	CBS 489.92^T^	KC146356	KP123973	KP124276	KP124895	KP125204	KP124740	KP124130
*A. ellipsoidialis*	MFLUCC 21-0132	MZ621989	OK236743	OK236690	OK236643	OK236596	–	–
*A. eupatoriicola*	MFLUCC 21-0122	MZ621982	OK236736	OK236683	OK236636	OK236589	–	–
*A. falcata*	MFLUCC 21-0123	MZ621992	OK236746	OK236693	OK236649	OK236599	–	–
*A. gaisen*	CBS 118488^R^	KP124427	KP123975	KP124278	KP124897	KP125206	KP124743	KP124132
*A. gossypina*	CBS 104.32^T^	KP124430	JQ646395	JQ646312	KP124900	KP125209	KP124746	KP124135
*A. herbiphorbicola*	CBS 119408^T^	KP124362	JQ646410	JQ646326	KP124830	KP125138	KP124673	KP124064
*A. iridiaustralis*	CBS 118486^T^	KP124435	KP123981	KP124284	KP124905	KP125214	KP124751	KP124140
*A. jacinthicola*	CBS 133751^T^	KP124438	KP123984	KP124287	KP124908	KP125217	KP124754	KP124143
*A. jingzhouensis*	YZU 221144^T^	OR883772	OR887694	OR887690	OR887688	OR887686	OR887684	OR887692
*A. kikuchiana*	CBS 107.53	KP124305	KP123858	KP124162	KP124774	KP125081	KP124613	KP124005
*A. koreana*	SPL-21^T^	LC621613	LC631831	LC621647	LC621681	LC621715	LC631857	LC631844
*A. lathyri*	MFLUCC 21-0140^T^	MZ621974	OK236728	OK236675	OK236628	OK236581	–	–
*A. lijiangensis*	YZU 221458^T^	OQ679970	OQ686781	OQ686785	OQ686789	OQ686783	OQ686787	OQ686779
*A. longipes*	CBS 121333^R^	KP124444	KP123990	KP124293	KP124914	KP125223	KP124761	KP124150
*A. longxiensis*	YZU 221221^T^	OQ534546	OQ473629	OQ512732	OQ543009	OQ512726	OQ543003	OQ512720
*A. longxiensis*	YZU 221222	OQ534547	OQ473628	OQ512731	OQ543008	OQ512725	OQ543002	OQ512719
***A. lycopersici***	YZU 221185^T^	OQ519795	OQ473633	OQ512736	OQ543013	OQ512730	OQ543007	OQ512724
	YZU 221186	OQ519794	OQ473632	OQ512735	OQ543012	OQ512729	OQ543006	OQ512723
	**YZU 241613**	**PX963241**	**PX975311**	**PX975325**	**PX975338**	**PX975377**	**PX975351**	**PX975364**
*A. macilenta*	MFLUCC 21-0138^T^	MZ621972	OK236726	OK236673	OK236626	OK236579	–	–
*A. macroconidia*	MFLUCC 21-0134^T^	MZ622001	OK236757	OK236704	OK236657	OK236610	–	–
*A. minimispora*	MFLUCC 21-0127^T^	MZ621980	OK236734	OK236681	OK236634	OK236587	–	–
*A. momordicae*	YZU 161378^T^	OR883774	OR887695	OR887691	OR887689	OR887687	OR887685	OR887693
*A. muriformispora*	MFLUCC 21-0784^T^	MZ621976	OK236730	OK236677	OK236630	OK236583	–	–
*A. myanmarensis*	YZU 231736^T^	OR897031	OR979657	OR963612	PP508256	OR963615	PP034184	OR979663
*A. oblongoellipsoidea*	MFLUCC 22-0074^T^	MZ621967	OK236721	OK236668	OK236621	OK236574	–	–
*A. obpyriconidia*	MFLUCC 21-0121^T^	MZ621978	OK236732	OK236680	OK236633	OK236585	–	–
*A. orobanches*	MFLUCC 21-0137^T^	MZ622007	OK236763	OK236710	–	–	–	–
*A. oryzicola*	YZU 231199^T^	PQ812549	PV155522	PV155536	PV155548	PV155528	PV155542	–
*A. ovoidea*	MFLUCC 21-0782^T^	MZ622005	–	OK236708	OK236661	OK236614	–	–
*A. phragmiticola*	MFLUCC 21-0125^T^	MZ621994	OK236749	OK236696	OK236649	OK236602	–	–
*A. platycodonis*	CBS 121348^T^	KP124367	KP123915	KP124219	KP124836	KP125144	KP124679	KP124070
*A. poae*	YZU 231197^T^	PQ812551	PV155524	PV155538	PV155550	PV155530	PV155544	PV155532
***A. pulvinifungicola***	CBS 194.86^T^	KP124316	KP123869	KP124172	KP124784	KP125092	KP124623	KP124016
	**YZU 221137**	**PX963234**	**PX975306**	**PX975320**	**PX975333**	**PX975372**	**PX975346**	**PX975359**
*A. rhadina*	CBS 595.93^T^	KP124320	JQ646399	KP124175	KP124788	KP125096	KP124627	KP124020
*A. rostroconidia*	MFLUCC 21-0136^T^	MZ621969	OK236723	OK236670	OK236623	OK236576	–	–
*A. salicicola*	MFLUCC 22-0072^T^	MZ621999	OK236753	OK236700	OK236653	OK236606	–	–
***A. sanguisorbae***	CBS 121456^T^	KP124369	KP123917	KP124221	KP124839	KP125147	KP124682	KP124073
	**YZU 171181**	**PX963226**	**PX975301**	**PX975314**	**PX975327**	**PX975366**	**PX975340**	**PX975353**
*A. septorioides*	CBS 175.80	KP124313	KP123866	JQ646324	KP124781	KP125089	KP124620	KP124013
***A. solanicola***	YZU 221189^T^	OQ534548	OQ473631	OQ512734	OQ543011	OQ512728	OQ543005	OQ512722
	YZU 221190	OQ519793	OQ473630	OQ512733	OQ543010	OQ512727	OQ543004	OQ512721
	**YZU 171596**	**PX963231**	**PX975305**	**PX975318**	**PX975331**	**PX975370**	**PX975344**	**PX975357**
*A. tomaticola*	CBS 118814^T^	KP124357	KP123906	KP124211	KP124825	KP125133	KP124669	KP124059
*A. tomato*	CBS 103.30	KP124445	KP123991	KP124294	KP124915	KP125224	KP124762	KP124151
*A. torilis*	MFLUCC 14-0433^T^	MZ621988	OK236741	OK236688	OK236641	OK236594	–	–
*A. vaccinii*	CBS 118818^T^	KP124359	KP123908	KP124213	KP124827	KP125135	KP124671	KP124061
*A. yamethinensis*	YZU 231739^T^	OR889008	OR979655	OR963610	PP179253	OR963614	PP034182	OR979661
*A. youyangensis*	YZU 234157	PP988497	PP860527	PP987153	PP869284	PP869286	PP869285	PP860528
***A. zeae***	YZU 231602^T^	PQ812548	PV155521	PV155535	PV155547	PV155527	PV155541	–
	**YZU 241610**	**PX963239**	**PX975309**	**PX975323**	**PX975336**	**PX975375**	**PX975349**	**PX975362**
	**YZU 241611**	**PX963238**	**PX975310**	**PX975324**	**PX975337**	**PX975376**	**PX975350**	**PX975363**

**Notes:** Strains obtained in this study are indicated in bold. Ex-type strains are marked with “T”, representative strains with “R”, and “–” indicates sequences that were unavailable.

## Data Availability

The nucleotide sequences generated in this study were deposited in GenBank database.
